# Preliminary design and evaluation of a remote tele-mentoring system for minimally invasive surgery

**DOI:** 10.1007/s00464-022-09164-3

**Published:** 2022-03-04

**Authors:** Dehlela Shabir, Nihal Abdurahiman, Jhasketan Padhan, Malek Anbatawi, May Trinh, Shidin Balakrishnan, Abdulla Al-Ansari, Elias Yaacoub, Zhigang Deng, Aiman Erbad, Amr Mohammed, Nikhil V. Navkar

**Affiliations:** 1 Department of Surgery, Surgical Research Section, Hamad General Hospital, Hamad Medical Corporation, PO Box 3050, Doha, Qatar; 2 Department of Computer Science, University of Houston, Houston, TX USA; 3 Department of Computer Science and Engineering, Qatar University, Doha, Qatar; 4 College of Science and Engineering, Hamad Bin Khalifa University, Doha, Qatar

**Keywords:** Minimally invasive surgery, Tele-mentoring, Augmented reality, Virtual surgical instruments, Telemedicine

## Abstract

**Background:**

Tele-mentoring during surgery facilitates the transfer of surgical knowledge from a mentor (specialist surgeon) to a mentee (operating surgeon). The aim of this work is to develop a tele-mentoring system tailored for minimally invasive surgery (MIS) where the mentor can remotely demonstrate to the mentee the required motion of the surgical instruments.

**Methods:**

A remote tele-mentoring system is implemented that generates visual cues in the form of virtual surgical instrument motion overlaid onto the live view of the operative field. The technical performance of the system is evaluated in a simulated environment, where the operating room and the central location of the mentor were physically located in different countries and connected over the internet. In addition, a user study was performed to assess the system as a mentoring tool.

**Results:**

On average, it took 260 ms to send a view of the operative field of 1920 × 1080 resolution from the operating room to the central location of the mentor and an average of 132 ms to receive the motion of virtual surgical instruments from the central location to the operating room. The user study showed that it is feasible for the mentor to demonstrate and for the mentee to understand and replicate the motion of surgical instruments.

**Conclusion:**

The work demonstrates the feasibility of transferring information over the internet from a mentor to a mentee in the form of virtual surgical instruments. Their motion is overlaid onto the live view of the operative field enabling real-time interactions between both the surgeons.

**Supplementary Information:**

The online version contains supplementary material available at 10.1007/s00464-022-09164-3.

Surgical tele-mentoring incorporates the use of information and telecommunications technology to transfer surgical knowledge from an expert surgeon (mentor) to an operating surgeon (mentee) [1–3]. The mentor and the mentee can be located physically apart at different geographical locations. Most of the current tele-mentoring systems in surgery involve exchange of audio, static annotations on the view of the operating field [4–6], and overlaid hand gestures displayed onto the operating field [7–9]. Although it is suitable for open surgeries, a more refined mechanism is required for Minimally Invasive Surgery (MIS). MIS involves the use of elongated surgical instruments and a scope inserted through small incisions to operate on the tissue. A tele-mentoring system developed for MIS should be able to demonstrate to the mentee, the interaction required between these articulated tooltips of the surgical instruments, and the tissue to be operated on. These cues would assist the mentee to visualize, comprehend, and perform the required surgical instrument movements. Thus, it would be relevant and helpful in different MIS tele-mentoring scenarios (as depicted in Table 1) to overlay motion of virtual surgical instruments onto the view of the operative field.

**Table 1:** Scenarios depicting application of tele-mentoring technology during MIS scenarios

MIS Scenario	Mentee	Mentor
Basic training for learning surgical skills	Surgical Fellow or Resident learning a surgical skill in simulation lab	An experienced instructor demonstrating the surgical skill
Transfer of skills to perform a new surgical method / procedure	A surgeon performing the surgical method / procedure for first time in an operating room	Specialist surgeon demonstrating the new surgical method / procedure
Providing guidance during a complicated surgery case	An expert surgeon performing the complicated surgery case in the operating room	Group of expert surgeons discussing the live surgery and providing feedback

The notion of using virtual surgical instruments’ motion overlaid onto the video of the operative field was first evaluated by Vera et al. [10] for teaching laparoscopic skills. The mentor uses a portable laparoscopic training box simulator with a green screen background and real surgical instruments (identical to the ones at the mentee’s site). Using the chromakey technique, the green screen in the background was filtered from the video and the motion of instruments controlled by the mentor was overlaid onto the live video of the operative field. Although the study demonstrated the potential of using overlaid virtual surgical tool motion for tele-mentoring, it requires the same setup at both the locations for the mentor and the mentee, thus making it inapplicable for intraoperative tele-mentoring during a surgery. It was also not feasible for robot-assisted MIS training scenarios as it would require manipulation of real robotic surgical instruments at the mentor site. Another study by Jarc et al. [11] demonstrated improvement in communication by displaying virtual surgical instrument motion (representing robotic surgical tooltips movement) in three dimensions during a training session between a mentor and a mentee. In a subsequent study [12], the same virtual instruments were used for realistic surgical tasks (tissue dissection and suturing in a live porcine model) re-emphasizing its effectiveness as a mentoring tool. However, the tele-mentoring studies [11, 12] were conducted using a standalone system, where both the mentor and the mentee were in the same room. A similar framework was proposed by Shabir et al. [13] for transferring the motion of virtual surgical instruments onto the operative field over a network. However, the prototype worked only on a local area network and the latency was significant to limit its usage for real-time guidance.

To prove efficacy for remote tele-mentoring, it would require (a) the mentor and the mentee to be connected on two systems located physically apart and (b) the mentor is able to demonstrate to the mentee and the mentee can understand the motion of the virtual surgical instruments overlaid onto live view of the operating field. The work presents an augmented reality-based system that facilitates remote tele-mentoring during a MIS. A remote tele-mentoring system compatible with manual (laparoscopic) as well as robotic surgical setup is proposed along with the surgical workflows followed by the mentor and the mentee. The technical details related to the implementation of the technology and experimental setup to evaluate the functioning of the system are described hereafter. The results show the functioning of the remote tele-mentoring system where the mentor and the mentee are located in different countries.

## Materials and methods

### Surgical workflow

**Fig. 1:**
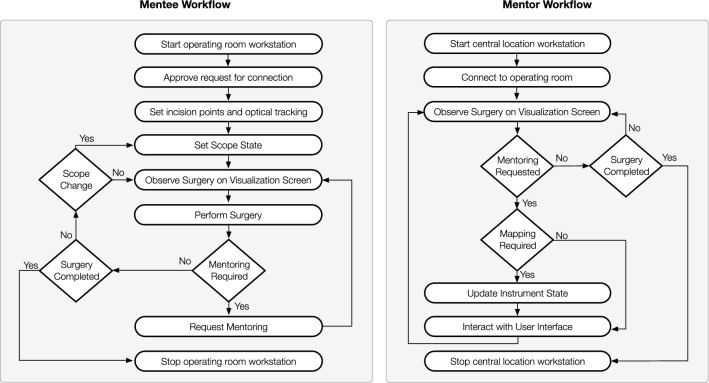
Workflow followed by the mentee and the mentor while using the tele-mentoring system for minimally invasive surgery. The mentor and mentee can be located geographically apart. The workstations in the operating room and at the central location need to be connected over a network

The proposed remote tele-mentoring system comprises two workflows (shown in Fig. 1). These workflows occur concurrently; one is governed by the mentee in the operating room and the other by the mentor. At the start of the MIS, the workstation at the mentor’s central location connects to the operating room workstation over a network. The mentee labels the incision points and configures the scope states (i.e., the scope angulation angle, field of view, and scope length) on the operating room workstation. An optical tracking system is used in the operating room to track the poses of the scope and positions of the incision points (via attaching tracking frames with optical markers to scope and trocars/cannulas). The information is also shared with the central workstation. The mentee visualizes the operative field on the screen and performs the surgery. The same view of the operative field is shared with the mentor. The system allows audio communication between the mentor and the mentee. When mentoring is required, the mentee requests the mentor’s involvement. The mentor updates the instrument state (i.e., maps virtual models of surgical instruments to the incision points and left-hand/right-hand user interface to control them). Then, the mentor demonstrates the required surgical instrument motion by manipulating virtual surgical instruments overlaid onto the live view of the operative field via the user interfaces. The motion of the virtual surgical instrument is transferred over the network and displayed in the operating room to the mentee. This acts as a visual cue and assists the mentee to perform the surgical sub-step required during the MIS using the remote tele-mentoring system.

### System architecture

**Fig. 2:**
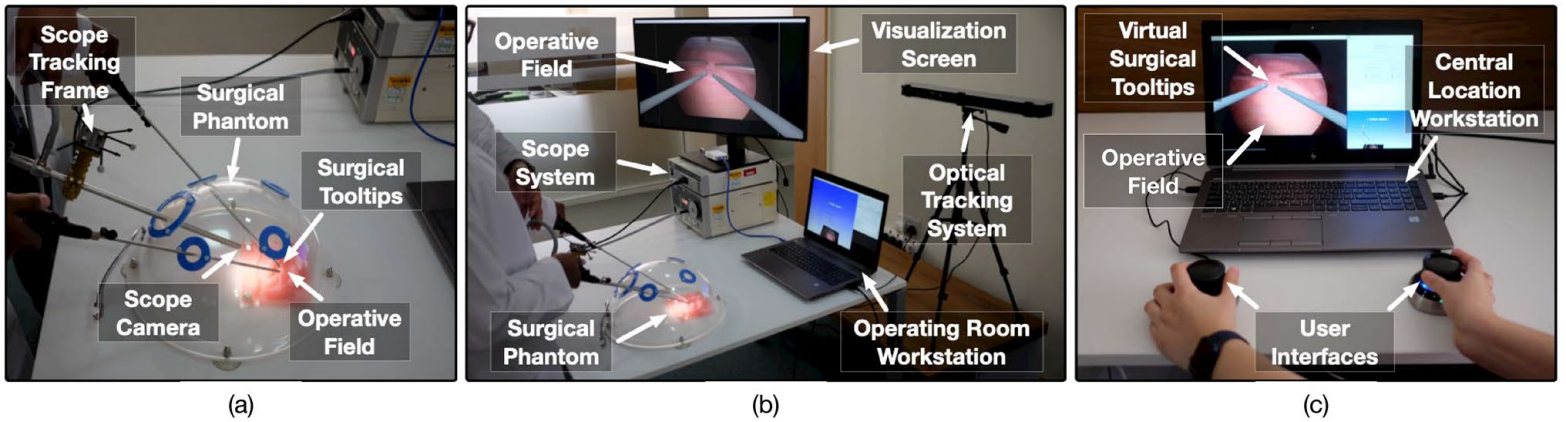
**a** A surgical phantom is used for laparoscopic/manual surgical setup. A 30-degree scope is used to capture the operative field and the motion of the surgical tooltips of laparoscopic instruments (needle drivers). A scope tracking frame is attached to the scope to track the pose of the scope camera. **b** An optical tracking system is used to track the pose of the scope tracking frame. The video of the operative field is bifurcated from the scope system to the operating room workstation. Operative field overlaid with virtual instruments received from the mentor’s workstation is displayed to the mentee on the visualization screen. **c** The mentor controls the motion of the virtual surgical instruments overlaid onto the live view of the operative field using the user interfaces on the mentor’s workstation

**Fig. 3:**
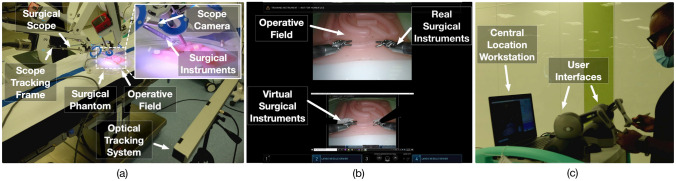
**a** The tele-mentoring system is integrated with a surgical robot (da Vinci Xi from Intuitive Surgical Inc.) and a surgical phantom is used for the robotic surgical setup. A 30-degree scope is used to capture the operative field and the motion of the surgical tooltips (EndoWrist needle drivers). A scope tracking frame is attached to the scope that enables tracking of the scope camera poses using the optical tracking system. **b** A tile-pro mode on the robot console is used to visualize the operative field and the operative field overlaid with the motion virtual instruments simultaneously. **c** The mentor controls the motion of the virtual surgical instruments using the user interfaces on the central location workstation

**Fig. 4:**
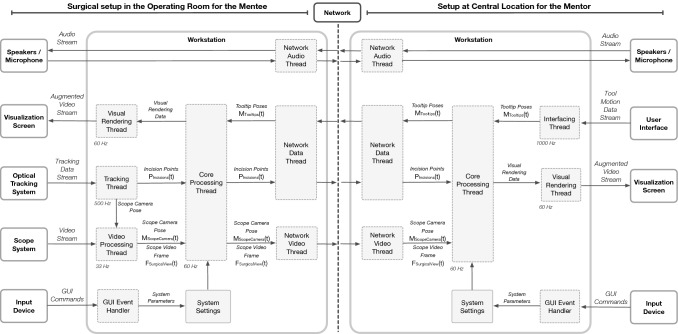
Architecture of the tele-mentoring system exhibiting interaction between the hardware and software components (running on workstations). The hardware units in the operating room and at the central location connects the mentee and the mentor over a network

The aforementioned workflow of tele-mentoring system was simulated for two minimally invasive surgical setups: (a) for manual MIS (Fig. 2) and (b) robot-assisted MIS (Fig. 3). For both the surgical scenarios, a common architecture of the tele-mentoring system (Fig. 4) was used. It consists of two primary setups, one centrally located with the mentor and the other inside the operating room with the mentee. The architecture is based on our previous work [13]. In the current work, two major improvements are made as compared to the previous architecture.

First, every interaction with the hardware unit (to process the data) is performed by a task-dedicated parallel-running thread. The multi-threaded architecture streamlines the flow of the processed data internally as well as externally with the hardware units and the network. This improves the overall performance of the tele-mentoring system. The processed data are described in Table 2 and its flow in the operating room and at the central location is governed by the core processing thread. At the operating room, the core processing thread (i) fetches the M_ScopeCamera_(t) and F_SurgicalView_(t) from the video processing thread, (ii) fetches P_Incisions_(t) from the tracking thread, (iii) sends the M_ScopeCamera_(t) and F_SurgicalView_(t) to the network video thread, (iv) sends P_Incisions_(t) to network data thread, (v) fetches M_Tooltips_(t) from network data thread, and (vi) send visual rendering data to be sent to visual rendering thread. Similarly, at the central location, the core processing thread (i) fetches M_Tooltips_(t) from the interfacing thread, (ii) send M_Tooltips_(t) to network data thread, (iii) fetches M_ScopeCamera_(t) and F_SurgicalView_(t) from network video thread, (iv) fetches P_Incisions_(t) from network data thread, and (v) send visual rendering data to be sent to visual rendering thread.

**Table 2:** Data processed and shared by the architecture of the tele-mentoring prototype

Data	Description of the processed data
Scope Camera Pose M_ScopeCamera_(t)	A 4 × 4 homogenous transformation matrix measured with respect to optical tracking system and representing the position and orientation of the scope’s camera at time instant ‘t.’ The tracking thread processes the tracking data stream acquired from the optical tracking system to extract the scope camera poses
Scope Video Frame F_SurgicalView_(t)	A frame of the operating field video at time instant ‘t.’ The video stream acquired from the scope system is processed by video processing thread to extract the video frame. It also combines the scope camera pose with scope video frame
Incision Points P_Incisions_(t)	A tuple storing the positions of the incision points at time instant ‘t’ and measured with respect to optical tracking system. Each element of the tuple represents an incision point. The tracking thread processes the tracking data stream acquired from the optical tracking system extract the incision points
Tooltip Poses M_Tooltips_(t)	A tuple storing left and right tooltip poses at time instant ‘t.’ Each element represents a co-ordinate frame in form of 4 × 4 homogenous transformation matrix attached to the tooltip of the augmented surgical instrument. The tool motion data stream acquired from the user interface is processed by the interfacing thread to extract tooltip poses
Visual Rendering Data	The data comprises of scope camera pose, scope video frame, incision points, tooltip poses, and system parameters. It is sent to the visual rendering thread, which uses the data to render scenes on visualization screen. The primary scene contains the augmented operative filed with overlaid virtual surgical tools. The secondary scene gives a 3D view of the surgical setup-assisting mentor to understand the configuration of incision points during surgery
System Parameters	The system parameters at the operating room workstation assists to set the labels to the incision points for intraoperative tracking, set the angulation angle of the scope, and accept the connection from the central location The system parameters at the central location workstation assists to set the network connection with the operating room and map virtual surgical tooltips to the incision points for left/right-hand tool movements

The second improvement is the integration of the WebRTC (Web Real-Time Communication) framework for networking instead of using the RTMP server. This integration facilitates tele-mentoring across geographical boundaries, drastically reduces the latency in sending/receiving the data over the network, and enables audio communication. Usage of WebRTC enables real-time communication capabilities and allows video, audio, and data to be exchanged between workstations in the operating room and at the central location. The networking threads are native to WebRTC. To establish a connection, the operating room workstation and the central location workstation discover their own public IPs using a STUN server (stun.l.google.com). A signaling server (hosted on DigitalOcean.com) is used to exchange the public IPs along with the media formats used by the networking threads. A direct peer-to-peer connection is established between the two workstations to initiate the communication required for the remote tele-mentoring.

### Experimental setup

#### Technical evaluation

To evaluate the technical performance of the developed remote tele-mentoring system within and across geographical boundaries, the system was tested under two modes of operation. In Mode I, the operating room workstation and the central location workstation were both located in the same city, Doha, Qatar. In Mode II, the operating room workstation was situated in Doha, Qatar, whereas the central location workstation was in Houston, Texas, USA. An Internet connection was used to connect both the workstations. The data sent and received by the networking threads on the workstations were logged and processed to evaluate the functioning of the tele-mentoring framework over the network.

The clocks on the workstations at the central location and at the operating room were synchronized from a common Network Time Protocol (NTP) server 216.239.35.4 (time2.google.com). The server synchronizes times among the connected workstations to a few milliseconds. However, because of asymmetric routes and network congestion, the time difference between the workstations and its NTP server clock may go off up to a few milliseconds. This difference was incorporated in the calculations to measure the clock drift between the operating room workstation and the central location workstation. The clock drift was added to the timestamps of the logged data to ensure synchronization between the clock of the operating room workstation and the clock of the central location workstation.

Performance under each mode was evaluated for different time durations (8, 10, 12 min). Each duration was evaluated for multiple trials (n = 3). The parameters to assess the performance included (i) delay in transferring information over the network, (ii) average time duration in receiving two consecutive data packets one-after-another over the network, (iii) degradation in the quality of the video frame caused by encoding–decoding, and (iv) drops in video frames over the network.

#### Evaluation as a mentoring tool

In addition to technical evaluation, a user study was conducted on the ease of using the remote tele-mentoring system for (a) mentor to visually demonstrate an instruction (in the form of virtual surgical instrument motion) and (b) for the mentee to understand and replicate mentor’s instructions. While previous user studies [10–13] have compared and demonstrated the advantage of using virtual surgical instruments motion over the static annotations and hand gestures in different surgical scenarios, this study focuses on the ease of generating and replicating the information provided by the mentor to the mentee.

**Fig. 5:**
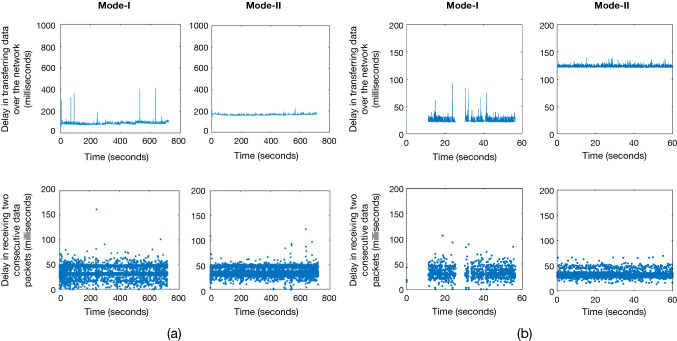
**a** The delay encountered in sending surgical scope camera poses M_ScopeCamera_(t) along with operating field video frame F_SurgicalView_(t) of 640✕ 480 pixels resolution from operating room to the central location workstation shown for a duration of 12 min. The delay in receiving two consecutive data packets comprising surgical scope camera poses M_ScopeCamera_(t) and operating field video frame F_SurgicalView_(t) at the central location workstation. **b** The delay encountered in sending virtual surgical instruments tooltip poses M_Tooltips_(t) from the central location workstation to the operating room workstation shown for a duration of one minute. The delay is not shown for the time period when there is no motion of the virtual surgical instruments. The delay in receiving two consecutive data packets comprising virtual surgical instruments tooltip poses M_Tooltips_(t) at the operating room workstation

The usability study was conducted with six mentor–mentee pairs (n = 6). Each pair performed 3 use cases. Therefore, a total of 18 use cases were evaluated. The mentors and mentees were selected from the surgical department at Hamad General Hospital, Qatar with at least a year experience in using the laparoscopic tools and Touch haptic device (by 3D Systems, USA). The study was approved by the institutional review board comprising the ethical committee (Medical Research Center, Doha, Qatar, approval number MRC-01–20-087). A research information sheet was presented to obtain the consent. In the study, both the mentor and the mentee were asked to perform a task simultaneously. The mentor moved the virtual surgical instrument tooltips along a predefined static path displayed on the central location workstation (Fig. 5a). The paths (n = 3) were represented by three-dimensional virtual curves. Each path corresponds to one use case. The motion of the virtual surgical instrument by the mentor was transferred over the network to the operating room workstation and was displayed onto the operative field (Fig. 5b). Mentee followed the displayed virtual surgical instrument motion using surgical instruments (laparoscopic needle driver).

During the study, the path demonstrated by the mentor and the path traversed by the mentee was recorded. At the central location, a Touch haptic device was used by the mentor to provide input for the motion of the virtual needle driver. The positions of the virtual instrument tooltip were logged. Similarly, at the operating room, the surgical instrument consists of a needle driver with an optical marker attached to its tooltip (Fig. 5c). An optical tracking system was used to track the position of the tooltip with respect to time, while the mentee moved the surgical instrument following the mentor’s instruction. The positions were processed to generate the trajectories traversed by the mentor (based on static-predefined paths) and by the mentee (based on the motion of the mentor).

## Results

### Technical performance

**Fig. 6:**
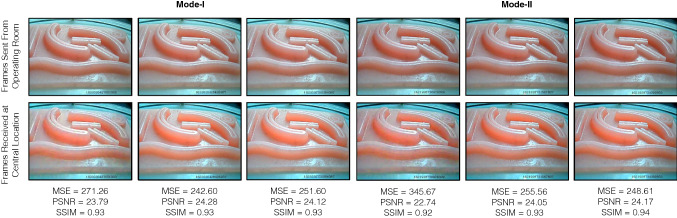
Visual comparison of the operating field video frame F_SurgicalView_(t) send by the operating room workstation before encoding and frames received at central location workstation after decoding in Mode I and Mode II of operation. The three-frame pair samples for each mode were selected randomly from the video stream

This section highlights the main findings of technical testing of the remote tele-mentoring system under the two modes of operation. The performance of the system is summarized in Table 3. It took relatively more time to send the information (comprising surgical operating field video frame along with surgical scope camera poses) across the network from the operating room to the central location in the case of Mode II as compared to Mode I (Fig. 6a). This was evident as in Mode II, the operating room and the central location were located geographically apart in two different continents. The delay for both modes were within the limit of 450 ms recommended by SAGES for live tele-mentoring [14]. The low latency also ensured that the mentor will be aware of the changes happening in the operating field during live surgery and can respond to any complications that may evolve intraoperatively in real time. As compared to the previous work [13], the latency was significantly reduced from 1560 to 163 ms (for 640 × 480 resolution video frames) and 260 ms (for 1920 × 1080 resolution video frames) for the transfer of information from the operating room to the central location. Also, the current system facilitated the transfer of information over the Internet across two countries as compared to a local area network in the same location.

**Table 3:** Performance of the remote tele-mentoring system under Mode I and Mode II

Description of the parameters		Mode I		Mode II	
Resolution of the operating field video frame sent from the operating room to the central location workstation		640 ✕ 480 pixels	1920 ✕ 1080 pixels	640 ✕ 480 pixels	1920 ✕ 1080 pixels
Average delay encountered in sending operating field video frame along with surgical scope camera poses from operating room to the central location workstation		78 ± 7 ms	115 ± 29 ms	163 ± 12 ms	260 ± 44 ms
Average delay in receiving two consecutive data packets comprising surgical scope camera poses and operating field video frame at the central location workstation		33 ± 27 ms	35 ± 12 ms	33 ± 6 ms	62 ± 54 ms
Percentage of the frames dropped when sent from the operating room workstation to the central location workstation		0.59%	0.70%	0.03%	8.34%
Video quality metric comparing frames sent before encoding by the operating room workstation and received after decoding by the central location workstation	Mean Square Error (MSE)	242.67	193.06	245.02	166.07
	Peak Signal-to-Noise Ratio (PSNR)	24.28	25.28	24.25	25.93
	Structure Similarity Index Measure (SSIM)	0.93	0.89	0.93	0.88
Average delay in transferring the surgical tooltips poses from the central location workstation to the operating room workstation		21 ± 2 ms		132 ± 23 ms	
Average time duration in receiving two consecutive data packets one-after-another from the central location workstation to the operating room workstation		26 ± 15 ms		33 ± 8 ms

While transferring the information from the central location to the operating room (primarily comprising virtual surgical tool motion in form of surgical tooltips poses), it took more time for Mode II as compared to Mode I (Fig. 6b). For both modes, this is within the limit of 200 ms proposed by Xu et al. [15] for tele-surgery. Another essential parameter while transferring the data from the operating room to the central location is the average time duration in receiving two consecutive data packets one-after-another. It ensured that the data are received at a uniform rate to the central location workstation and the operating room workstation.

**Fig. 7:**
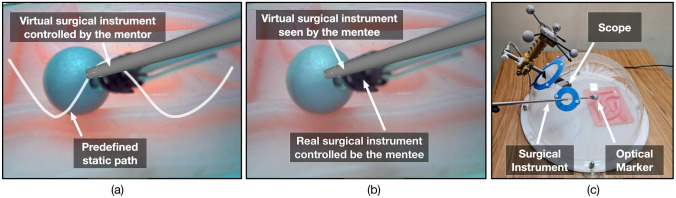
**a** Setup for the user study. An optical marker is attached to the surgical instrument tooltip to track its position using the optical tracking system. **b** Surgical field displayed on central location workstation. The mentor follows the predefined static path using the virtual surgical instrument. **c** Surgical field displayed on operating room workstation. The mentee tries to replicate the motion of the virtual surgical instrument using a real surgical instrument

Standard video quality metrics were used to analyze the distortion of the surgical video caused by encoding and decoding over the network. More emphasis was given on SSIM (as compared to PSNR and MSE) as it has been proved to be more correlated to the quality of perception of the human visual system [16, 17]. SSIM compares luminance, contrast, and structure to model image distortion, whereas PSNR and MSE have an inability to discriminate structural content in images [16, 17]. The SSIM of 0.93 for 640 × 480 pixels resolution and the SSIM of 0.88 for 1920 × 1080 resolution is practical for the mentor to understand the operating field. Video frame samples received at the central location are presented in Fig. 7.

### Mentoring capabilities

**Fig. 8:**
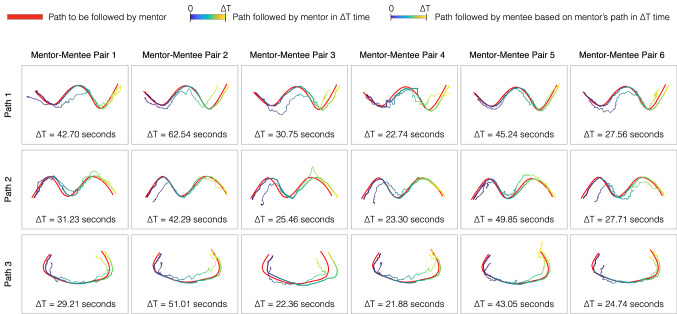
Paths followed by the mentor and the mentee during the usability study. The static-predefined path displayed on the central location workstation to the mentor is shown in red color. The path followed by mentor and mentee is shown using a color map that represents temporal relation for the duration of the task from 0 to ∆T s. The operating field is of size 64.66 × 48.49 mm^2^

While the aforementioned section showed the technical feasibility of the remote tele-mentoring system, this section discusses the feasibility of using the system as a surgical mentoring tool. That is, whether is it feasible for the mentee to understand the instructions provided by the mentor. The outcomes of the user study are presented in Fig. 8 and Table 4. To compare the paths traversed by the mentor and the mentee in the same space, the paths were projected on a 2D viewing plane (60 mm apart from the scope’s camera) with a width and height of 64.66 mm and 48.49 mm. The viewing plane represented the operative field. The scope’s camera horizontal field of view was 35˚ with an aspect ratio of 4:3. Figure 8 presents the projected paths on the viewing plane for each task performed by the mentor–mentee pair. Table 4 shows the measured parameters for the user study tasks corresponding to the three paths. The parameters used were based on previous studies [18, 19] for assessment of surgical skills and included (a) average duration to complete the task by the mentor–mentee pair, (b) similarity measure between two paths expressed as Dynamic Time Warping (DTW) distance [20], (c) average distance between two paths computed as DTW distance divided by the number of sample points on the curve to be aligned, and (d) Fréchet distance between two paths (which is the shortest distance sufficient to follow-up a point moving on one curve with a point moving on the other curve in same direction) [21].

**Table 4:** Comparison of the paths defined by surgical instrument’s tooltip motion

Description of parameters		Path-1	Path-2	Path-3	Path average
Average duration for mentor and mentee to complete the task		38.59 ± 14.62 s	33.30 ± 10.50 s	32.04 ± 12.16 s	34.65 ± 12.14 s
Measures to compare the path generated by the mentor with the static-predefined path	DTW distance	1235.9 ± 300.7	1139.5 ± 360.3	1154.2 ± 500.7	1176.5 ± 331.8
	Average distance	1.19 ± 0.19 mm	1.26 ± 0.37 mm	1.40 ± 0.88 mm	1.28 ± 0.45 mm
	Fréchet distance	2.52 ± 0.61 mm	2.40 ± 1.18 mm	2.80 ± 1.90 mm	2.57 ± 1.09 mm
Measures to compare the path generated by the mentor and the mentee during the user study	DTW distance	3625.2 ± 1680.6	2900.8 ± 812.5	3059.9 ± 881.8	3195.3 ± 971.4
	Average distance	3.38 ± 0.93 mm	3.19 ± 0.56 mm	3.60 ± 1.15 mm	3.39 ± 0.76 mm
	Fréchet distance	7.25 ± 1.32 mm	7.80 ± 1.11 mm	8.28 ± 1.53 mm	7.78 ± 1.02 mm

While traversing path-1, path-2, or path-3, no significant difference was found in the DTW distance for the mentor to follow static-predefined path and mentee to follow mentor’s path. Irrespective of the shape, the mentor was able to follow the predefined path with an average distance of 1.28 ± 0.45 mm and Fréchet distances of 2.57 ± 1.09 mm. This shows it is feasible for a mentor to demonstrate the required tool–tissue interaction during a surgical sub-step by generating a visual cue in the form of a virtual surgical instrument motion overlaid onto the live view of operative field. Similarly, for all the paths, the mentee was also able to replicate the mentor’s virtual surgical instrument motion with an average distance of 3.39 ± 0.76 mm and Fréchet distances of 7.78 ± 1.02 mm. As the mentee was able to replicate the motion, it is possible for the mentee to understand in real time the motion of the virtual surgical instrument controlled by the mentor. This is shown in Fig. 8 by the color-coded paths representing spatial–temporal relation.

It should be noted that it may not be practical during a surgery for the mentee to continuously follow the motion of the virtual surgical instruments to perform the required tool–tissue interaction. In an ideal surgical tele-mentoring scenario, the mentor needs to first demonstrate the required interaction virtually between the surgical tooltip and the tissue using the virtual surgical instrument (along with audio cues). At the operating room, by analyzing these audio and visual cues on the operating field, the mentee needs to mentally grasp the required motion in context with the tissue to be operated and then perform it after the mentor has demonstrated the surgical sub-step.

## Discussion

The remote tele-mentoring system facilitates real-time guidance from a mentor to a mentee during an MIS, who are physically located apart. The guidance is in the form of audio-visual cues. The visual cues comprise virtual surgical instrument motion overlaid onto the live view of the operative field. The multi-threaded architecture and integrated WebRTC framework reduce the latency and ensure synchronization between the augmented data streams. This allows the mentor to demonstrate to the mentee, the tool–tissue interaction required during a MIS.

The current system has certain limitations. One of the limitations of the proposed system is the usage of an optical tracking system. The line-of-sight of the optical tracking system may get restricted during an MIS by the surgical team members standing close to the operating table. It may also be ineffective for single incision MIS due to the close placements of trocars [22, 23]. Also, surgeries through natural orifices with articulated scopes and instruments [24–26] may need a mechanism to track the exit points of the endo-luminal cannulas. In such cases, an electromechanical tracking system may be useful to triangulate the poses and compute the incision points. Another drawback of our current system is that any increase in the video resolution could compromise the seamless transfer of the surgical video during tele-mentoring. Although video quality up to HD (1920✕1080 pixels) is reasonable for transmission, any further increase (for example ultra-HD) is not suitable using the current system. Thus, it limits the usage of the system and may need integration of adaptive video streaming protocols as per network bandwidth [27, 28]. Lastly, conceptual frameworks and learning theories suited for the system need to be developed [29, 30]. As per the user study, a structured method needs to be designed for effective communication between the mentor and the mentee. A standardized lexicon/protocol would be vital to ensure smooth communication. This would require conducting further user studies to understand the communication between mentor–mentee for different surgical scenarios.

The proposed real time-augmented reality-based system of overlaying virtual surgical instruments is expected to support and further enhance the conventional collaborative methods (static annotations on the view of the operating field [4–6] and overlaid hand gestures [7, 8, 31]). The additional generated visual cues can be used by the mentor to discuss and advice on general intraoperative sub-steps (similar to existing methods). Under the assumption that both mentor and mentee have comparable surgical macro-skills (such as general expertise in anatomy, maneuvering of surgical instruments, ability to identify surrounding critical structures, and judge tissue thickness), the proposed system is primarily expected to be helpful in scenarios where the mentor remotely guides a less experienced mentee in performing a newly developed surgical technique. The mentee may not have perfected the technique-relevant micro-skills (such as visual tactility, economy of movement, and tissue handling [32, 33]) and the overlaid virtual surgical instruments may expedite the learning. Additionally, since standard operating procedures do not currently exist for tele-collaborative surgical initiatives, the proposed system of augmentation could assist in establishing correlation between taxonomy and surgical tool movements. Further relevant user experience studies among mentor–mentee need to be conducted to assess the impact of the proposed method on surgical tele-collaboration in MIS.

Tele-mentoring poses unique challenges from a medicolegal perspective. Legal requirements for medical licensing as well as associated surgical privileges vary nationally and globally. For situations where there exists no physician–patient relationship, courts have ruled that informal physician consults cannot be considered malpractice [34]. When the mentor merely advices a mentee, it is considered as a consultation where there exists no relationship between the mentoring physician and the patient. In such a case, the mentor does not require a medical license at the treating site/facility for informal consultation as the mentee who is the primary medical authority on-site assumes all medical liabilities [34–36]. However, according to the Society of American Gastrointestinal and Endoscopic Surgeons (SAGES), teleconsultation and tele-mentoring are considered different, and although the mentee is considered competent, in tele-mentoring the mentor is still equally responsible in providing care to the patient [1]. Furthermore, in the USA and Canada, meeting medical licensing requirements in one state or province does not imply eligibility to practice in another state, with exceptions like Delaware and West Virginia where inter-state eligibility is allowed. On the contrary, there are also positive precedents, like the lower legal restrictions in the European Union, where a licensed physician has the privilege to practice anywhere else in the European Union [37], which sets a good example for other countries to follow. Thus, for effective utilization of remote tele-mentoring, there needs to be further global ratification of introducing flexible laws concerning international medical licensing requirements, and medical liability considerations should be addressed before the procedure through clear communication between the mentor, the mentee, and the patient.

The future work would be geared toward four fronts. First, we plan to modify and extend the proposed tele-mentoring system for open surgeries. It would require modification of underlying networking framework (WebRTC) to transfer additional information (such as depth map acquired from RGB-D cameras). This information, pertaining to open surgery operative field, can be rendered in an immersive environment on a virtual reality display for the mentor [38]. On the other hand, a head-mounted display can be used to render dynamic holograms of virtual surgical instruments motion onto the view of the mentee [39–41]. Second, there is potential for the software modules of the proposed tele-mentoring system to be integrated with existing commercial platforms [5, 31, 42]. This could further enhance the commercial systems by augmenting the information with overlaid virtual surgical instrument motion. Third, we plan to assess the system under 5G network’s Ultra-Reliability and Low-Latency Communications (URLLC) use case. URLLC corresponds to certain communication services that can be considered critical and are intolerant to delay. It may allow transmitting 4 K videos, while partially reducing latency, especially at relatively short distances. It should be noted that such advanced services depend on the deployment of adequate infrastructure. Such infrastructure is available mostly in urban areas [43], but significant connectivity gaps exist between these areas and the rural areas of developing countries [44]. Thus, even with 5G deployments, the current networking framework will still be useful in regions where the state-of-the-art technologies are not yet deployed. Lastly, before first-in-human studies, multisite animal studies will be required to assess the working of the proposed system in a minimally invasive setting (manual and robotic). It would further assist in understanding the functioning of the tele-mentoring system in an operating room environment, especially related to ergonomics of the hardware components used at the operative field (such as tracking frames for trocars and line-of-sight of the optical tracking system) and interaction with the software graphical user interfaces [45–47].

## Supplementary Information

Below is the link to the electronic supplementary material.

Supplementary file1 (DOCX 72 KB)
