# Supplementary material for: Preliminary design and evaluation of a remote tele-mentoring system for minimally invasive surgery
Source: Surg Endosc. 2022 Mar 4;36(5):3663–74. doi: 10.1007/s00464-022-09164-3 (PMC9001542; doi:10.1007/s00464-022-09164-3)
Supplement: Supplementary file 1 — Supplementary file1 (DOCX 72 KB) [file 464_2022_9164_MOESM1_ESM.docx]

| **Terms** | **Description of the terms used in the context of the manuscript** |
| --- | --- |
| Virtual | A real-world object simulated or displayed using a computer. |
| Augmented reality | Technology that generates a composite view by superimposing a computer-generated image on a user's view of the real world. |
| Computer thread | A single sequence of a set of instructions that can execute independently. In the proposed architecture, multiple threads are executed concurrently and communicate with each other. |
| IP address | IP refers to "Internet Protocol". It is a set of rules that governs the format of data sent/received over the network. IP address is a unique address that identifies a device on the network. It contains locations information of the device on the internet. Thus, it allows the devices to be located and connected over internet. |
| Server | On a computer network, a central computer that provide service to other computers (clients). |
| User interface | Medium which humans uses to interact with the computers. It could be hardware devices that can be used to provide a set of input commands to a computer by a user. |
| Data packet | Data (a set of values) that has been made into a single package and sent over a network from one computer to other. |
| Video frame | One of the still image in a video. Combining and playing the video frames one after another, creates the effect of a moving picture. |
